# Deep learning-based breast cancer diagnosis in breast MRI: systematic review and meta-analysis

**DOI:** 10.1007/s00330-025-11406-6

**Published:** 2025-02-05

**Authors:** Kamarul Amin Abdullah, Sara Marziali, Muzna Nanaa, Lorena Escudero Sánchez, Nicholas R. Payne, Fiona J. Gilbert

**Affiliations:** 1 Department of Radiology, University of Cambridge School of Clinical Medicine, Cambridge Biomedical Campus, Cambridge, UK; 2 Universiti Sultan Zainal Abidin, Terengganu, Malaysia; 3 Department of Radiology and Radiotherapy, Istituto Nazionale dei Tumori, Milan, Italy; 4 Cancer Research UK Cambridge Centre, Li Ka Shing Centre, Cambridge, UK; 5 Department of Radiology, Addenbrookes Hospital, Cambridge University Hospitals NHS Foundation Trust, Cambridge, UK

**Keywords:** Artificial intelligence, Deep learning, Breast neoplasms, Magnetic resonance imaging, Meta-analysis as topic

## Abstract

**Objectives:**

The aim of this work is to evaluate the performance of deep learning (DL) models for breast cancer diagnosis with MRI.

**Materials and methods:**

A literature search was conducted on Web of Science, PubMed, and IEEE Xplore for relevant studies published from January 2015 to February 2024. The study was registered with the PROSPERO International Prospective Register of Systematic Reviews (protocol no. CRD42024485371). The quality assessment of diagnostic accuracy studies-2 (QUADAS2) tool and the Must AI Criteria-10 (MAIC-10) checklist were used to assess quality and risk of bias. The meta-analysis included studies reporting DL for breast cancer diagnosis and their performance, from which pooled summary estimates for the area under the curve (AUC), sensitivity, and specificity were calculated.

**Results:**

A total of 40 studies were included, of which only 21 were eligible for quantitative analysis. Convolutional neural networks (CNNs) were used in 62.5% (25/40) of the implemented models, with the remaining 37.5% (15/40) hybrid composite models (HCMs). The pooled estimates of AUC, sensitivity, and specificity were 0.90 (95% CI: 0.87, 0.93), 88% (95% CI: 86, 91%), and 90% (95% CI: 87, 93%), respectively.

**Conclusions:**

DL models used for breast cancer diagnosis on MRI achieve high performance. However, there is considerable inherent variability in this analysis. Therefore, continuous evaluation and refinement of DL models is essential to ensure their practicality in the clinical setting.

**Key Points:**

***Question*** *Can DL models improve diagnostic accuracy in breast MRI, addressing challenges like overfitting and heterogeneity in study designs and imaging sequences*?

***Findings*** *DL achieved high diagnostic accuracy (AUC 0.90, sensitivity 88%, specificity 90%) in breast MRI, with training size significantly impacting performance metrics (p* *<* *0.001)*.

***Clinical*** ***relevance*** *DL models demonstrate high accuracy in breast cancer diagnosis using MRI, showing the potential to enhance diagnostic confidence and reduce radiologist workload, especially with larger datasets minimizing overfitting and improving clinical reliability*.

## Introduction

Continuous efforts for early detection and improved treatment are essential in the fight against breast cancer. Recent statistics from the UK Office for National Statistics show a persistently high age-standardized mortality rate for breast cancer (34.1 per 100,000 women in 2016) [1]. Many countries have added ultrasound to the standard mammography in women with increased breast density in their screening programs with annual MRI used for women at very high risk. Women should be screened, based upon recommendations with a frequency varying depending on the level of risk.

Breast MRI is becoming increasingly important in screening programs, especially for women at very high risk. Following the publication of the DENSE trial, the European Society of Breast Imaging (EUSOBI) guidelines recommended breast MRI as an additional screening tool for women with dense breast tissue [2–4]. This shift emphasizes the growing importance of MRI for earlier and more accurate detection of breast cancer. Given the increased complexity and data volume of multislice images compared to 2D mammography, the interpretation of breast MRI images relies heavily on the radiologist’s expertise. With the growing demand for breast MRI examinations, the workload has increased considerably [5, 6], leading to longer waiting times and possible delays in diagnosis [7, 8]. In this context, artificial intelligence (AI) is proving to be a promising tool to support radiologists by improving the accuracy and speed of breast MRI interpretation.

Recent studies show that AI can improve clinical decision-making and patient outcomes by analyzing large datasets to identify areas of concern, potentially reducing both false-positive and false-negative results [9, 10]. Deep learning (DL) models, a subset of machine learning (ML) AI, have significantly changed the field of medical imaging in recent years. Known for their ability to process unstructured data and gain insights autonomously without manual feature extraction, DL models have made remarkable advances in tasks such as classification and segmentation. The application of DL in cancer detection has been extensively studied for mammography [11] and various other cancer types [12], as well as for clinical image analysis more broadly [13]. Recent investigations have also shown that DL plays an important role in improving breast MRI performance for cancer diagnosis [14, 15]. This systematic review synthesizes the latest developments and analyzes the diagnostic performance of DL models on MRI datasets in breast cancer diagnosis. Supplemental Text 1 (online) contains a glossary of terms.

## Materials and methods

This systematic review and meta-analysis were reported in accordance with the preferred reporting items for a systematic review and meta-analysis of diagnostic test accuracy studies guidance [16]. The review protocol was registered with the PROSPERO International Prospective Register of Systematic Reviews (no. CRD42024485371) (Supplemental Text 2 [online]). Data generated or analyzed during the study are available from the corresponding author by request.

### Literature search

Digital literature databases, including Web of Science, PubMed, and the Institute of Electrical and Electronics Engineers (IEEE), were searched for publications from January 1, 2015, to February 1, 2024, to include the latest advancements in DL and increased data availability. This period was chosen since the earliest reported use of DL in breast MRI was in 2016 [17]. The initial search was conducted by K.A.A., who has more than three years of experience with meta-analyses. The search strategy and keywords were refined with the co-reviewers (S.M. and M.N.), who are breast radiologists with more than two years of experience. Manual searches of included article references were also conducted for the same period. The keywords used for this search were: “Deep Learning”, “Deep Neural Network*”, “DNN”, “Convolutional Neural Network*”, “CNN”, “Artificial Intelligence”, “AI”, “Machine Learning”, “Breast Cancer”, “Breast Carcinoma”, “Breast Tumor*”, “Breast Tumor*”, “Breast Neoplasm*”, and “Magnetic Resonance Imaging”, “MRI”.

### Study selection and data extraction

All original articles included in this systematic review and meta-analysis were retrospective studies that met predefined inclusion and exclusion criteria. The inclusion criteria comprised studies that: (i) focused on breast cancer diagnosis; (ii) were relevant to AI and DL, and (iii) applied DL methods for diagnostic purposes. Studies were excluded if they: (i) did not utilize MRI as the imaging modality; (ii) did not employ DL algorithms; (iii) focused solely on segmentation tasks; (iv) provided insufficient or inappropriate data; and (v) studies related only to radiomics or radio genomics. Studies were required to report at least three diagnostic accuracy metrics namely, area under the receiver operating characteristic curve (AUC), sensitivity, and specificity.

The screening process involved two independent reviewers (K.A.A. and S.M.) initially screening titles and abstracts, followed by a full-text review of eligible studies. Discrepancies were resolved by a third reviewer (M.N.) at each stage. Reasons for exclusions were systematically documented to ensure transparency.

Data extraction was performed using a custom-designed spreadsheet to standardize the process. Extracted data included: (i) study-level information (study ID, article number, publication year); (ii) performance metrics (AUC, sensitivity, specificity, accuracy, and their respective confidence intervals); (iii) study characteristics (sample size, number of MRI examinations, type of MRI sequences used, training-validation-test splits, and whether the study was single-center or multi-center); and (v) methodological details (preprocessing techniques, network types, data augmentation, k-fold cross-validation, and use of public datasets).

Regular team meetings were conducted to discuss discrepancies and ensure consistent application of the eligibility criteria.

### Quality assessment and risk of bias

The risk of bias and quality assessment of all included studies were rigorously assessed using the quality assessment of diagnostic accuracy studies-2 (QUADAS-2) tool [18] by two reviewers (K.A.A. and S.M.), with disagreements resolved by a third reviewer (M.N.). Additionally, the Must AI Criteria-10 (MAIC-10) [19] checklist was implemented to evaluate the design and reporting of studies on AI in medical imaging. MAIC-10, a more concise checklist, has been shown to have high coherence with the Checklist for AI in Medical Imaging (CLAIM) [20, 21] while simplifying the assessment process. The conceptual alignment between MAIC-10 and CLAIM ensures that the critical elements of CLAIM are inherently covered by MAIC-10’s streamlined checklist. This approach allowed for a practical yet robust evaluation of the included studies while maintaining consistency with widely accepted reporting standards. The combined use of QUADAS-2 for assessing methodological quality and risk of bias, and MAIC-10 for AI-specific reporting, ensured a balanced and comprehensive quality assessment.

### Meta-analysis protocol and statistical analysis

Two complementary approaches were adopted to address the inherent heterogeneity in the extracted data. The first involved a meta-analysis of studies reporting the three primary diagnostic accuracy metrics: AUC, sensitivity, and specificity. Subgroup analyses and meta-regressions were then conducted to investigate factors contributing to heterogeneity.

The subgroup analysis considered several variables, including: (i) the type of DL used (CNNs or hybrid composite models [HCMs]); (ii) the level of assessment (lesion, slice, or patient); (iii) the type of MRI sequences used (dynamic contrast-enhanced MRI (DCE-MRI), mpMRI, T1W, T2-weighted sequences (T2W) or DWI); (iv) the study design (single center vs multi-center); (v) the dimensions of images (2D, 3D or both) input to the DL; and (vi) the training size categories of small (< 300 samples), medium (301–600 samples) and large (> 600 samples). These categories were based on typical training sizes seen in similar studies, facilitating meaningful comparisons, and identifying trends in model performance across different scales of training data.

Meta-regression was performed to assess whether training dataset size was a significant source of heterogeneity in model performance. Pooled estimates of diagnostic accuracy metrics, including 95% confidence intervals (CIs), were calculated using a random-effects model to account for variability between study outcomes [21]. Forest plots were generated to visualize pooled estimates for AUC, sensitivity, and specificity. Heterogeneity among studies was evaluated using the Q-test (*p* < 0.05 indicating significant heterogeneity) and quantified using the *I*² statistic. *I*² values were categorized as: not important (0–25%), low (26–50%), moderate (51–75%), and high (76–100%) heterogeneity [22, 23].

All statistical analyses were conducted with R version 4.3.3 with R Studio version 2023.12.1. The ‘meta’ package (version 7.0-0) was specifically used for performing meta-analyses and generating pooled proportion estimates. Statistical significance was set at α = 0.05.

## Results

### Study selection and data extraction

**Fig. 1:**
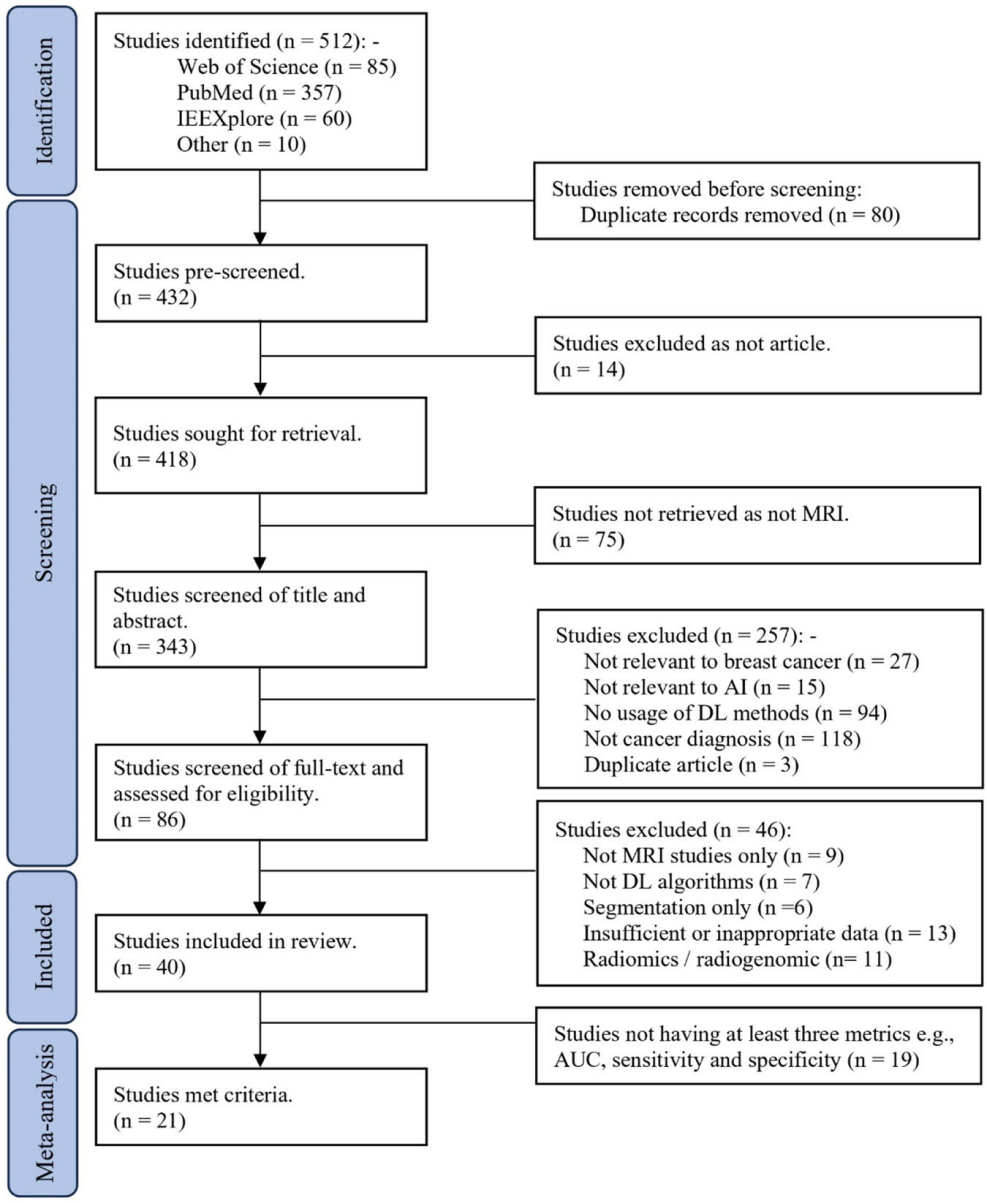
Preferred reporting items for systematic reviews and meta-analyses (PRISMA) flow diagram of screened and included papers. MRI, magnetic resonance imaging; IEEE, Institute of Electrical and Electronics Engineers; AI, artificial intelligence; DL, deep learning; AUC, area under the ROC curve

A preferred reporting item for a systematic review and meta-analysis (PRISMA) diagram (Fig. 1) demonstrates the process of study inclusion. The search of electronic literature databases and hand searching returned 512 studies. After removing duplicates, non-article formats, and studies not related to MRI, 343 studies remained. Screening of titles and abstracts excluded 257 studies, leaving 86 full-text articles for review. Of these, 40 articles were included in the qualitative review (references of the included studies can be found in Table S1 [online]). Out of the 40, 21 provided all performance metrics of AUC, sensitivity, and specificity and were included in the quantitative meta-analysis.

**Fig. 2:**
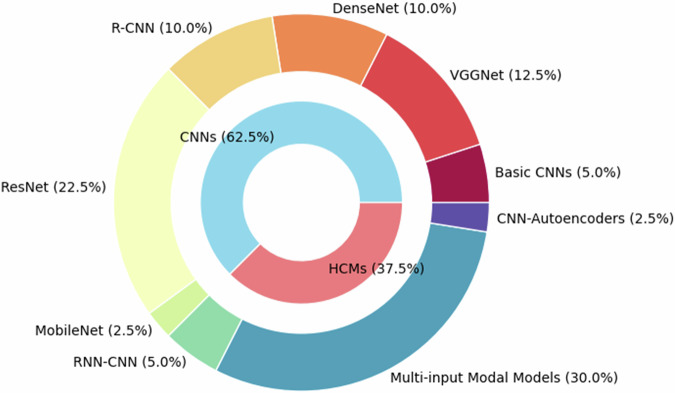
DL models distribution including their main and subcategories proposed in breast MRI research for cancer diagnosis

**Fig. 3:**
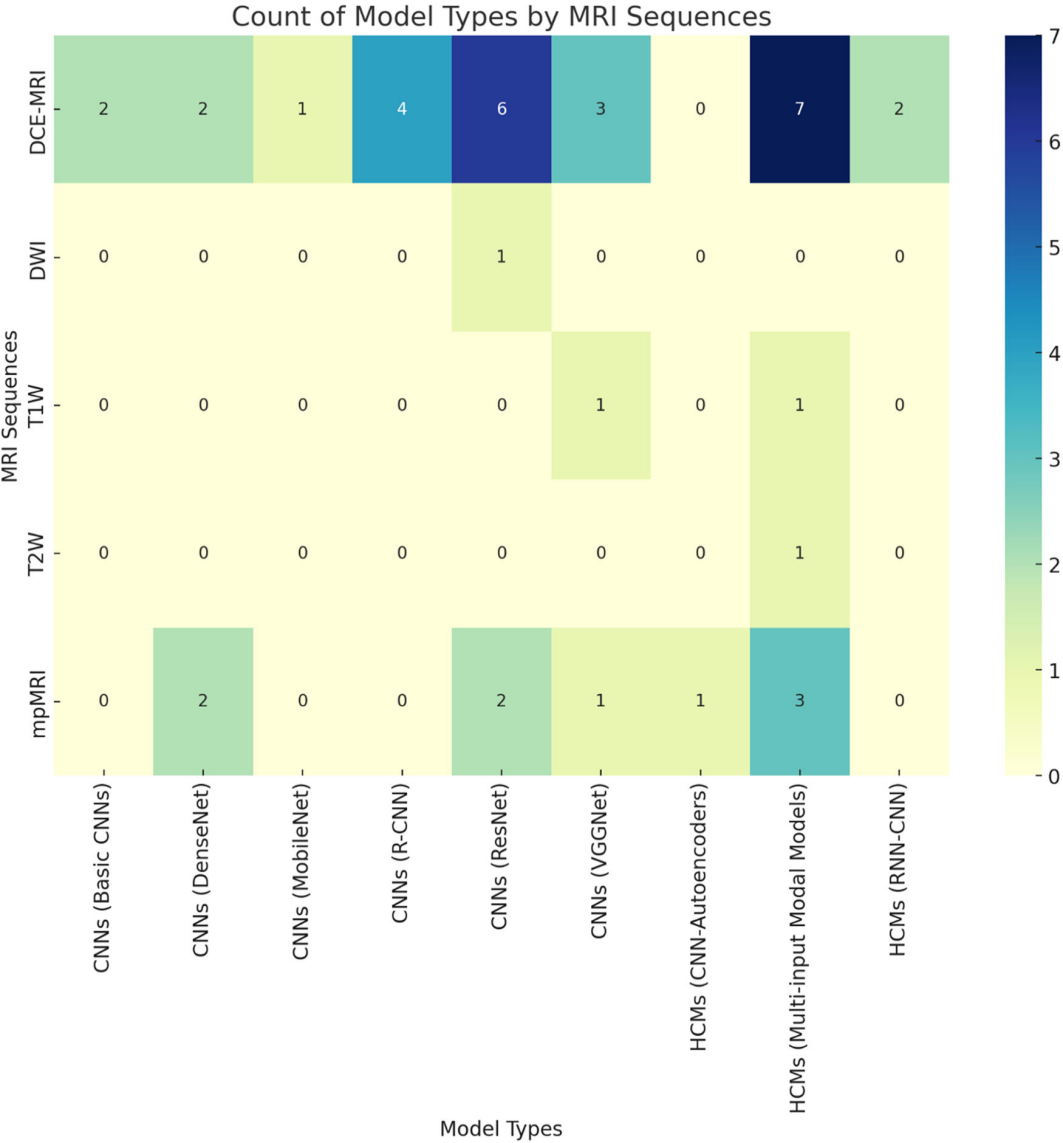
Relationship between MRI sequences (*y*-axis) and DL networks (*x*-axis) for cancer diagnosis in breast MRI interpretation based on DL models. DCE, dynamic contrast-enhanced; mpMRI, multi-parametric magnetic resonance imaging; T1W, T1-weighted; DWI, diffusion-weighted imaging; T2W, T2-weighted; AE, autoencoder; CNNs, convolutional neural networks; RCNN, region-based CNN; RNN, recurrent neural network

The studies included were published between 2017 and 2024, demonstrating significant progress in this field in recent years. Of the 40 included studies, 62.5% (25/40) implemented CNNs [24], while the remaining 37.5% (15/40) used HCMs. ResNet [25] and VGGNet [26] were the most commonly used CNN models, while RNN-CNN [27] and multi-input modal models were the primary types for HCMs (Fig. 2). Five types of MRI sequences were selected for input to the DL models (Fig. 3). DCE-MRI and multiparametric MRI (mpMRI) sequences were used most frequently, followed by T1-weighted (T1W), diffusion-weighted (DWI), and T2W sequences. Table 1 categorizes the DL models identified in the included studies, subdividing them into main categories, subcategories, and specific networks.

**Table 1:** Types of DL including the main categories, subcategories and their specific networks used in the study for all 40 included studies

Main category and subcategory	Specific networks
CNNs	
VGGNet	ConvNet VGGNet [41], VGGNet-19 [14, 40, 42, 43]
Basic CNNs	ZFNet [33], Dual-Branch CNN [62]
ResNet	ResNet-101 [29, 30], ResNet-50 [34, 37, 39], 3D-ResNet [15, 35], Modified ResNet [36, 38]
R-CNN	Faster R-CNN [63, 64], 3D RetinaNet [65], RetinaNet [66]
MobileNet	MobileNetV1, MobileNetV2 [67]
DenseNet	3D DenseNet19 [57], DenseNet201 [28], 3D DenseNet/DCNN [68, 69]
HCMs	
RNN-CNN	VGGNet19 + LSTM [70], YOLOv5x + LSTM [71]
Multi-input modal models	UNet + VGG16 [54], U-Net + 3D CNN [47], CNN + AE + CPGANFS [48], U-Net +++ Faster RCNN + ResNet-101 [55], ResNet-18 + LSTM [45], 3D U-Net + ResNet-34 [46], CNN ensembles [44], ResNet + 3D-CBAM [49], RCNN + YOLACT [50], FMS-PCNN + SU-Net [51], Mask R-CNN + ResNet-50 [52], ResNet-50 + LSTM [53]
CNN-Autoencoders	SSAE + SVM [31]

Table 2 provides a comprehensive overview of the dataset distribution, performance metrics, preprocessing, and validation techniques across the 21 included studies. Sample sizes varied considerably, with patient cohorts ranging from 56 to 3426. Training sizes averaged 158 patients for small datasets, 445 patients for medium datasets, and 2150 patients for large datasets. Reported AUC values ranged from 0.71 to 0.99, sensitivity values from 58% to 98%, and specificity values from 66% to 96%. Cross-validation techniques, particularly *k*-fold validation, were widely employed. Data split ratio, including data management strategies such as the use of one center as an independent testing dataset in a multicenter [28–31], ensured that testing data remained entirely unseen during training. This approach provided a robust assessment of model generalizability.

**Table 2:** A comprehensive overview of the 21 studies included in our quantitative meta-analysis, which evaluates DL algorithms for diagnosing breast cancer using MRI

Authors (year)	Sample size (*n* of patients)	Total data split (training size [*T*], validation size [*V*], testing size [*Ts*]) for DL Input*	Training size category**	Data split ratio (testing dataset: internal/external)	Total no. of lesions (*n*)***	Performance metrics (AUC/sensitivity/specificity/accuracy)	Cross-validation (*k*-fold)	Public dataset (yes/no)	Preprocessing (segmentation type/other methods)	Data augmentation (yes/no)
Adachi et al 2020 [54]	371	214 (T):72 (V):85 (Ts) (patients)	Small	3:1 (internal)	Total: 371 (50 [N], 89 [B], 232 [M])	0.93 (95% CI: 0.88, 0.97)/0.93/0.83/not stated	Not stated (hold-out validation)	No (single center)	Converted to jpeg format and magnified six times (resizing)	Yes
Antropova et al 2019 [65]	703	9600 (T + V):2400 (Ts) (images)	Large	8:2 (not stated)	Total: 703 (221 [B], 482 [M])	0.88/0.90/0.58/not stated	Not stated (hold-out validation)	No (not stated)	Region of interest (ROI) were created (manual segmentation)	No
Chen et al 2022 [60]	364	Not stated	Not stated	Not stated (internal)	Total: 364 (130 [B], 234 [M])	0.96/0.96/0.79/0.90	5-fold	No (not stated)	Lesion localization and stacking for 3D volumes. (Manual segmentation)	Yes
Cong et al 2024 [35]	694	218 (T):73 (V):278 (Ts) (patients)	Small	Not stated (internal and external) (holding out one center as an independent dataset)	Total: 1944 (835 [N], 481 [B], 628 [M])	0.94 (95% CI: 0.89, 0.97)/0.89/0.90/not stated	Not stated	Both (multicenter)	Cropping, denoising, resizing, and normalization with z-scoring (cropping and normalization)	Not stated
Eskreis-Winkler et al 2021 [36]	273	217 (T):27 (V):29 (Ts) (patients)	Small	8:1:1 (internal)	Not stated	0.95 (95% CI: 0.94, 0.97)/0.90 (95% CI: 0.87, 0.92)/0.94 (95% CI: 0.93, 0.96)/0.93 (95% CI: 0.90, 0.94)	5-fold	No (not stated)	Selecting central 50% of slices containing tumors (cropping)	Yes
Fan et al 2023 [41]	246 (S)	172 (T):37 (V):37 (Ts) (patients)	Small	Not stated (not stated)	Total: 247 (140 [B], 107 [M])	0.87 (95% CI: 0.78, 0.95)/0.94/0.76/0.84	10-fold	No (not stated)	Breast area segmented excluding chest wall with skin removed (manual segmentation)	Not stated
Feng et al 2020 [57]	100	60 (T):20 (V):20 (Ts) (Patients)	Small	6:2:2 (not stated)	Total: 100 (32 [B], 68 [M])	0.91/0.86/0.85/0.86	Random sub-sampling	No (not stated)	Pixel-level segmentation by radiologists (manual segmentation)	Yes
Gui et al 2022 [55]	337	277 (T):90 (V + Ts) (lesions)	Small	Not stated (not stated)	Total: 337 (171 [B], 166 [M])	0.96/0.95/0.90/0.93	Not stated	No (single center)	Segmentation based on labeling levels (not stated)	Not stated
Hizukuri et al 2021 [29]	56 (S)	38 (T):18 (V + Ts) (patients)	Small	2:1 (internal)	Total: 56 (30 [B], 26 [M])	0.95/0.93/0.92/0.93	3-fold	No (single center)	Determined baseline CNN with Bayesian optimization (model optimization)	Yes
Hu et al 2021 [30]	1979	1164 (T):291 (V):535 (Ts) (lesions)	Large	8:2 (internal)	Total: 1990 (lesions) (496 [B], 1494 [M])	0.93 (95% CI: 0.91, 0.96)/0.94/0.72/not stated	Not stated	No (not stated)	Feature-level and image-level analysis (not stated)	No
Liu et al 2022 [44]	438	60,037 (T):15,010 (V):13,024 (Ts) (images)	Large	8:1:1 (external) (holding out one center as an independent dataset)	Not stated	0.92 (95% CI: 0.86, 0.98) / 0.74 (95% CI: 0.58, 0.91) / 0.95 (95% CI: 0.89, 1.00) / 0.94 (95% CI: 0.88, 1.00)	Not stated	Yes (multicenter)	MRI volumes were normalized and cropped (cropping and normalization)	No
Parekh et al 2020 [68]	195	145 (T):50 (V + Ts) (patients)	Small	Not stated (internal and external) (holding out one center as an independent dataset)	Not stated	0.90 (95% CI: 0.84, 0.94)/0.86/0.86/not stated	10-fold	Both (multicenter)	Hybrid 3D wavelet and affine transformation for multiparametric registration (registration)	Not stated
Rasti et al 2017 [31]	112	Not stated	Not stated	Not stated (internal)	Total: 562 (318 [B], 244 [M]	0.99/0.98/0.95/0.96	5-fold	No (not stated)	Automatic ROIs were used (automatic egmentation)	No
Sheng et al 2021 [72]	203	145 (T):22 (V):40 (Ts) (patients)	Small	7:1:2 (internal)	Total: 207 (101 [B], 106 [M])	0.83/0.79/0.78/0.79	5-fold	No (single center)	Segmentation of breast. (automatic segmentation)	Not stated
Tang et al 2023 [47]	203	145 (T):22 (V):40 (Ts) (patients)	Small	Not stated (internal)	Total: 207 (101 [B], 106 [M])	0.89/0.80/0.86/0.83	5-fold	No (single center)	Intensity normalized between 0 and 1. (normalization)	Not stated
Wang et al 2022 [42]	903	482 (T):121 (V):150 (Ts) (Set A)/211 (Ts) (Set B) (lesions)	Medium	4:1 (internal)	Total: 852 (539 [B], 426 [M])	0.82 (95% CI: 0.76, 0.87)/0.85 (95% CI: 0.75, 0.93)/0.66 (95% CI: 0.58, 0.74)/0.73 (95% CI: 0.66, 0.78)	Not stated	No (single center)	Cropped MIP resized to 768 × 768. (cropping and resizing)	Yes
Yin et al 2021 [67]	802	420 (T):140 (V):242 (Ts) (patients)	Medium	3:1 (internal and external)	Not stated	0.85 (95% CI: 0.77, 0.93)/0.82/0.83/0.82	Not stated	No (single center)	Polygon ROIs of lesions cropped and reshaped to 128 x 128 x 22. (cropping and resizing)	Yes
Yin et al 2023 [34]	319	190 (T):62 (V):67 (Ts) (patients)	Small	Not stated (internal)	Total: 319 (165 [B], 154 [M])	0.94 (95% CI: 0.88, 1.00)/0.93/0.95/0.94	5-fold	No (not stated)	Geometric transformations; blocks centered on lesion cropped and reshaped to 224 × 224 × 3. (cropping and resizing)	Yes
Zhang et al 2022 [15]	339	241 (T):98 (V + Ts) (patients)	Small	Not stated (external) (holding out one center as an independent dataset)	Not stated	0.71/0.80/0.74/0.75	10-fold	No (multicenter)	Fuzzy C-means tumor segmentation on subtraction images. (automatic segmentation)	Not stated
Zhou et al 2019 [51]	1537	1073 (T):157 (V):307 (Ts) (patients)	Large	Not stated (internal)	Total: 1537 (506 [B], 1031 [M])	0.86/0.91 (95% CI: 0.86, 0.94)/0.69 (95% CI: 0.60, 0.78)/0.84 (95% CI: 0.79, 0.87)	Not stated	No (single center)	Subtracted T1W, applied morphological processing, and stacked 2D slices to obtain 3D, Gaussian smoothing. (image processing)	Not stated
Zhu et al 2022 [33]	3426	2456 (T):728 (V + Ts) (lesions)	Large	Not stated (internal and external)	Total: 3743 (1449 [B], 2294 [M])	0.89 (95% CI: 0.84, 0.92)/0.73 (95% CI: 0.66, 0.88)/0.88 (95% CI: 0.80, 0.94)/0.79 (95% CI: 0.74, 0.84)	Not stated	No (not stated)	Not stated	Yes

^* ^*T* training, *V* validation, *Ts* testing

^** ^*S* training size = 600

^*** ^*N* normal, *B* benign, *M* malignant

### Quality assessment

**Fig. 4:**
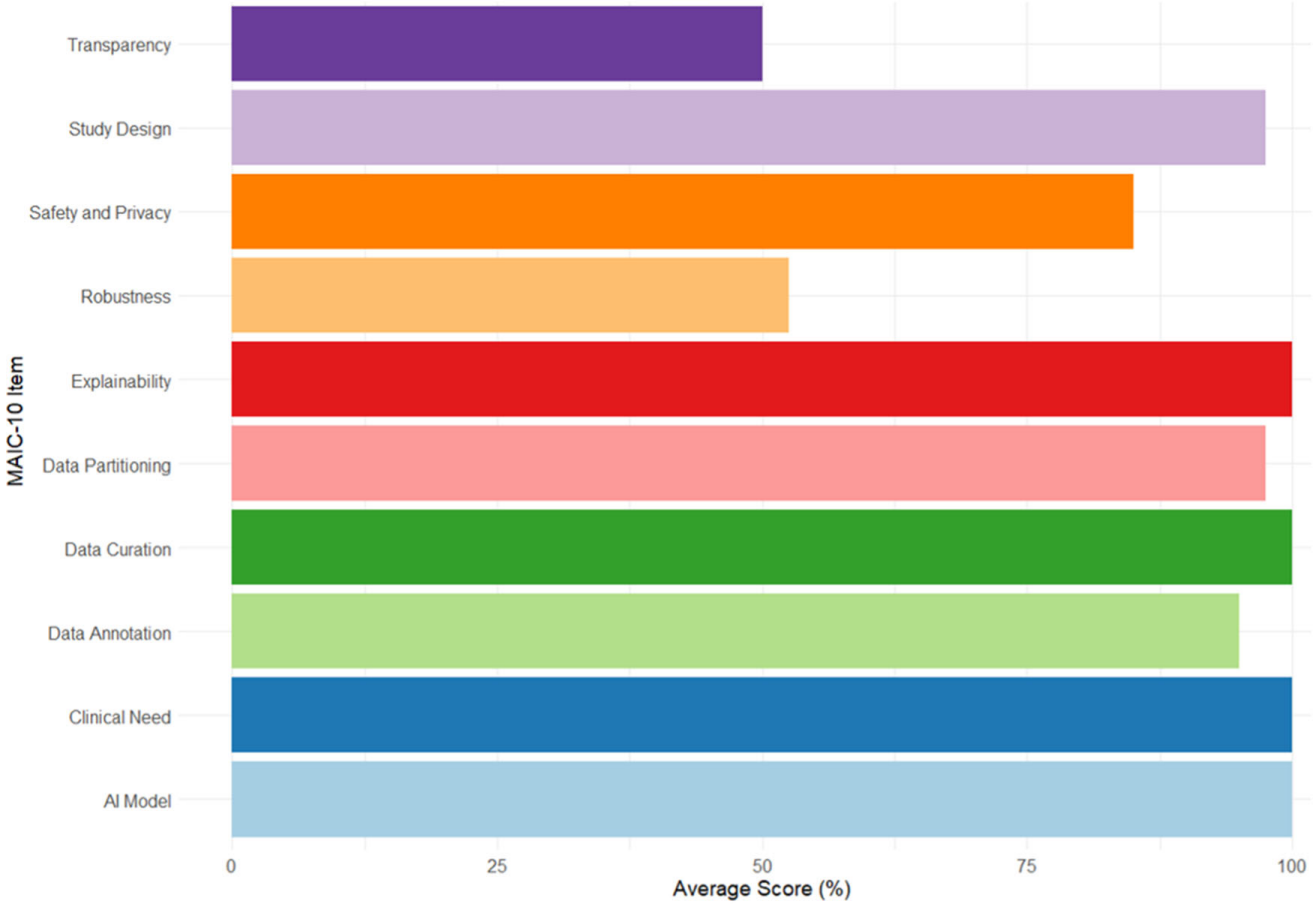
The graph represents the scores for the MAIC-10 quality assessment items related to AI studies, expressed as a percentage. The scores are based on the assessment of the 40 different studies, and the items focus on various aspects including clinical need, study design, safety and privacy, data curation, data annotation, data partitioning, AI model, robustness, explainability and transparency

**Fig. 5:**
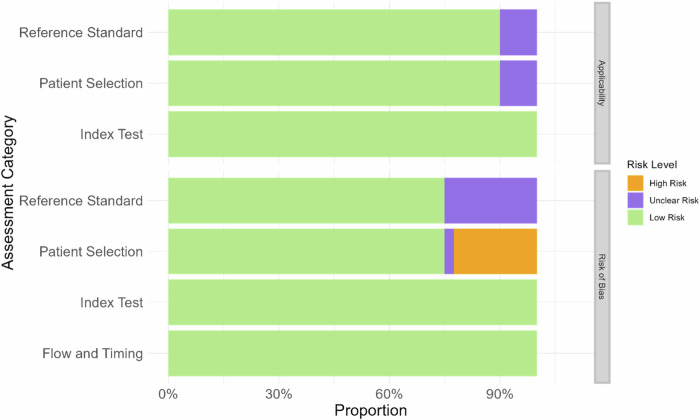
A visual summary of the quality assessment for the 40 studies included in our analysis, according to the guidelines of the QUADAS-2 tool. Each horizontal bar segment corresponds to the proportion of studies in a particular assessment category, shown as a percentage of the total

The Quality Assessment of Diagnostic Accuracy Studies 2 (QUADAS-2) tool was applied to all included studies in this review, with summary results shown in Fig. 4 and Tables S2 and S3 (online). This tool identified a high risk of bias for analysis, as well as significant applicability concerns for the index test, participants, and patient selection (Fig. 4). In the patient selection category, 75% of studies were low risk, 22.5% were high risk and 2.5% were unclear. All studies in the index test category were rated as low risk. The reference standard category had 75% low risk and 25% unclear. The MAIC-10 checklist bar graph (Fig. 5) shows high average scores for ‘clinical need’ (100%), ‘study design’ (97.5%), ‘security and privacy’ (85%), ‘data curation’ (100%), ‘data annotation’ (95%), ‘data partitioning’ (97.5%), ‘AI model’ (100%), and ‘explainability’ (100%). However, ‘robustness’ (52.5%) and ‘transparency’ (50%) scored lower, indicating the need for improvements for reliable AI clinical applications. Overall, the studies demonstrated low bias, high applicability, and good quality, despite some high-risk ratings.

### Assessment of performance metrics

Overall results show high performance, with pooled estimates of 0.90 for AUC (95% CI: 0.87, 0.93), 88% for sensitivity (95% CI: 86, 91%), and 90% for specificity (95% CI: 87, 93%). Considerable heterogeneity was found between studies, confirmed by Cochrane *Q*-tests (AUC: *Q* = 138.99, *p* < 0.01; sensitivity: *Q* = 44.40, *p* < 0.01; specificity: *Q* = 95.93, *p* < 0.01) and increased Higgins *I*² statistics (AUC: *I*² = 85.6%; sensitivity: *I*² = 55%; specificity: *I*² = 79.2%). Contributions of individual studies to these metrics are visualized in the forest plots (Figs. S1 and S3 [online]).

Table 3 presents the results of the subgroup and meta-regression analyses. The analysis showed no statistically significant differences in performance between the categories examined. Despite considerable heterogeneity (*I*²: 87.92% for AUC, 63.53% for sensitivity, and 93.08% for specificity), the type of DL models (CNNs vs HCMs) did not significantly impact diagnostic performance. Similarly, the type of MRI sequences used (DCE-MRI, mpMRI, T1W, T2W, and DWI) did not significantly affect performance metrics, despite consistently high heterogeneity (*I*²: 87.01% for AUC, 55.47% for sensitivity, 89.86% for specificity).

**Table 3:** The table presents the results of the subgroup and meta-regression analyses, including the type of DL models, MRI sequences, assessment levels, study designs, image dimensions, and training sizes

Subgroup	AUC (95% CI)	Sensitivity (95% CI)	Specificity (95% CI)	*I*²	*Q*	*p-*value for heterogeneity	*p*-value for moderator
Type of DL models (CNN vs HCM)	0.89 (0.86, 0.93)	88% (85%, 91%)	83% (77%, 89%)	87.92%	138.96	< 0.0001	0.5304
MRI sequences (DCE-MRI, mpMRI, etc.)	0.90 (0.86, 0.93)	90% (86%, 93%)	79% (74%, 85%)	87.01%	112.15	< 0.0001	0.8272
Assessment levels (lesion, slice, etc.)	0.90 (0.85, 0.94)	88% (83%, 92%)	80% (73%, 87%)	87.63%	128.03	< 0.0001	0.9848
Study designs (single-center vs multi-center)	0.87 (0.81, 0.93)	82% (76%, 88%)	87% (79%, 94%)	87.01%	84.58	< 0.0001	0.7118
Image dimensions (2D, 3D, or both)	0.89 (0.86, 0.93)	87% (84%, 91%)	82% (76%, 89%)	87.55%	129.67	< 0.0001	0.8676
Training size (total number)	0.86 (0.79, 0.93)	91% (87%, 95%)	69% (58%, 80%)	53.98%	4.35	0.1139	< 0.0001

Assessment levels (lesion, slice, image, or patient) also showed no effect on performance, indicating the robustness of the DL models across different scales of observation. Study designs (single center vs multi-center) and image dimensions (2D, 3D, or both) showed high heterogeneity but no significant impact on performance metrics. However, the total number of training sizes had a significant effect on performance metrics. The overall effect was significant for AUC (QM (d*f* = 15) = 44.6739, *p* < 0.0001), sensitivity (QM (d*f* = 15) = 36.0487, *p* = 0.0017), and specificity (QM (d*f* = 15) = 60.3986, *p* < 0.0001). Further analysis of training size categories also revealed significant differences in diagnostic performance for small (< 300), moderate (300–600), and large (> 600) training sizes, with considerable heterogeneity in AUC (*I*² = 86.70%). AUC values were consistently high across categories, while sensitivity and specificity showed significant estimates with moderate and high heterogeneity (*I*² = 54.62% and *I*² = 90.74%), respectively. Training size categories significantly impacted performance metrics, with all *p-*values below 0.0001 (Table 4).

**Table 4:** The table shows the results for the subgroup analyses for AUC, sensitivity, and specificity based on training size categories

Metric	Pooled estimate (95% CI)	Standard error (SE)	*Z*-value	*p*-value for moderator	*I*²	*Q*	*p*-value for heterogeneity
AUC							
Small < 300	0.90 (0.87, 0.93)	0.02	50.98	< 0.0001	86.70%	112.99	< 0.0001
Moderate 301–600	0.83 (0.74, 0.92)	0.05	18.06	< 0.0001			
Large > 600	0.90 (0.84, 0.95)	0.03	33.30	< 0.0001			
Sensitivity							
Small < 300	88% (85%, 92%)	0.02	50.58	< 0.0001	54.62%	32.76	0.0079
Moderate 301–600	84% (75%, 92%)	0.04	18.78	< 0.0001			
Large > 600	87% (82%, 93%)	0.03	30.90	< 0.0001			
Specificity							
Small < 300	86% (80%, 91%)	0.03	29.90	< 0.0001	090.74%	173.99	< 0.0001
Moderate 301–600	75% (61%, 89%)	0.07	10.46	< 0.0001			
Large > 600	77% (68%, 86%)	0.04	17.10	< 0.0001			

## Discussion

This systematic review and meta-analysis showed that DL models are highly effective in detecting breast cancer by MRI, with pooled estimates showing an AUC of 0.90, a sensitivity of 88%, and a specificity of 90%. These findings highlight the robustness of DL models across varied imaging conditions and clinical settings, demonstrating their potential for integration into routine diagnostic workflows. Subgroup analyses did not reveal significant differences in performance across key factors, including the type of DL architecture, MRI sequences, image level assessments, image dimensions, and study design (single-center vs multicenter). However, training size significantly influenced performance, with smaller datasets potentially contributing to overfitting. These results reflect the adaptability of DL models but also indicate the need for larger, high-quality datasets and external validation.

CNNs dominated the included studies, accounting for 62.5% (25/40) of the models analyzed. Their widespread adoption can be attributed to their powerful feature extraction capabilities and extensive research history [32, 33]. Among CNN architectures, ResNet (38%) [15, 29, 30, 34–39] and VGGNet (21%) [14, 40–43] were the most commonly employed, consistently delivering high-performance metrics. However, CNNs have limitations in modeling long-range dependencies and sequential data. HCMs, which accounted for 37.5% (15/40) of the models, addressed these limitations by integrating CNNs for spatial processing and RNNs for sequential data. Multi-input modal models, comprising 75% of HCMs [44–55] demonstrated significant potential by incorporating multimodal data to enhance diagnostic accuracy. This trend toward hybrid approaches reflects a shift in research focus toward improving versatility and robustness.

DCE-MRI was the most frequently used sequence (68%, 27/40) due to its superior contrast resolution and ability to visualize tumor vascularity. Studies such as Antropova et al [41] showed its effectiveness, achieving an AUC of 0.88. The combination of T2W and DCE-MRI (mpMRI) further improved diagnostic performance, as shown by Parekh et al [31], which achieved an AUC of 0.90. Feature-based methods, particularly employing subtracted maximum-intensity projection (MIP), demonstrated superior diagnostic accuracy compared to image fusion methods [56]. These findings suggest that integrating multiple sequences or using advanced preprocessing techniques may enhance DL model performance.

No significant differences in performance were observed across image-level assessments, whether at the lesion, image, or patient level. This suggests that DL models are adaptable to various input levels. However, variability in how studies defined and implemented these assessments highlights the need for standardized protocols to ensure consistency and reproducibility in future research. Similarly, no significant differences were observed between 2D, 3D, or mixed image dimensions. This finding reflects the flexibility of DL models in processing data of varying complexity. However, 3D inputs may offer additional contextual information, particularly for volumetric imaging, suggesting an avenue for further research.

Multicenter studies are expected to provide more robust validation due to their inherent diversity. Four multicenter studies [28–31] reserved one center exclusively as the testing dataset, ensuring that the testing data remained entirely unseen during training. This approach strengthens generalizability by simulating novel clinical settings. However, the lack of significant differences in performance between single-center and multicenter studies suggests that other factors, such as data quality and preprocessing, may play a more critical role in influencing performance. The nine studies that did not report dataset management practices further highlight the importance of transparency in study design.

Nevertheless, training sizes significantly influenced model performance. Smaller datasets (< 300 patients) demonstrated higher performance, potentially due to overfitting. For example, Hizukuri et al (2021) [33] reported an AUC of 0.95 with only 38 training patients. In contrast, larger datasets (> 600 patients), as used by Zhou et al [57], achieved lower but more generalizable metrics (AUCs 0.86). These findings highlight the trade-off between dataset size and performance. While small datasets may yield high accuracy, they risk poor generalizability [58]. Larger datasets, although more challenging to manage, provide a more reliable foundation for clinical implementation.

The findings of this meta-analysis also demonstrate that DL models for breast MRI diagnosis achieve performance metrics comparable to or exceeding those reported for radiologists. For example, Warren et al [56] reported a sensitivity of 88.6% and specificity of 69.2% among radiologists, with significant interobserver variability, while Narayanan et al [59] found sensitivities of 82% and specificities of 67% even among experienced radiologists. Training and standardization have been shown to improve radiologist performance, as evidenced in multicenter trials. A meta-analysis by Peters et al [60] reported a sensitivity of 90% and specificity of 72%, emphasizing the impact of variability in lesion criteria and cancer prevalence, while Mussurakis et al [61] highlighted observer variability in interpreting DCE-MRI patterns. In contrast, the DL models analyzed in this study demonstrated consistent and high performance (AUC 0.90, sensitivity 88%, specificity 90%) across imaging sequences and study designs. These findings suggest that DL models could complement radiologists by reducing interobserver variability and improving diagnostic reliability, though further studies directly comparing DL models with radiologists are warranted.

This meta-analysis study had several limitations. The retrospective nature of the included studies introduces potential challenges including variability in protocols, data completeness, and potential bias inherent to retrospective data collection. It also focused exclusively on breast cancer diagnosis using MRI, despite significant advances in other areas such as molecular type classification, histopathological assessment, prediction of response to neoadjuvant chemotherapy, and lymph node metastases detection. Additionally, the study did not thoroughly address pre-processing techniques, which are critical because errors in normalization, augmentation, and segmentation can significantly affect model performance and generalizability. The inherent variability of the DL algorithms used across studies, including differences in complexity and architecture between CNNs and HCMs, posed challenges for direct comparison and impacted diagnostic accuracy. Furthermore, the study did not fully investigate the effects of different MRI parameters and scanner types, which may significantly influence image quality and the performance of DL models. While metrics such as the structural similarity index measure (SSIM), and peak signal-to-noise ratio (PSNR) provide some insight into image quality, there remains a need for standardized assessment methods tailored to breast MRI. The quality assessment and risk of bias tools employed in this study were limited to QUADAS-2 and MAIC-10. Although these tools complement each other effectively, their scope may not fully address all challenges unique to AI-specific methodologies in DL research. Additionally, while cross-validation techniques were commonly employed, incomplete reporting of specific training-validation-test splits in some studies limits reproducibility and transparency. Most studies relied on internal dataset splits for testing, which may introduce bias and overestimate model performance due to homogeneity between training and testing datasets. Finally, while multicenter studies were included, only four explicitly reported holding out one center as an independent testing dataset, limiting our ability to assess the robustness of their findings.

This meta-analysis demonstrates the adaptability and robustness of DL models for breast cancer detection using MRI. While DL models maintained high performance under varied conditions, the findings emphasize the importance of dataset quality, transparent reporting, and external validation to enhance generalizability. Future studies should prioritize standardized imaging protocols, multicenter datasets, and multimodal imaging approaches to further improve the clinical applicability of DL models.

## Supplementary information

ELECTRONIC SUPPLEMENTARY MATERIAL

## Data Availability

Data generated or analyzed during the study are accessible upon request from the corresponding author.

## Abbreviations

AI: Artificial intelligence
AUC: Area under the receiver operating characteristic curve
CNNs: Convolutional neural networks
DCE-MRI: Dynamic contrast-enhanced MRI
DL: Deep learning
DWI: Diffusion-weighted
HCMs: Hybrid composite models
ML: Machine learning
mpMRI: multiparametric MRI
T1W: T1-weighted
T2W: T2-weighted sequences
