# Supplementary material for: Deep learning-based breast cancer diagnosis in breast MRI: systematic review and meta-analysis
Source: Eur Radiol. 2025 Feb 5;35(8):4474–89. doi: 10.1007/s00330-025-11406-6 (PMC12226709; doi:10.1007/s00330-025-11406-6)
Supplement: Supplementary file 1 — ELECTRONIC SUPPLEMENTARY MATERIAL [file 330_2025_11406_MOESM1_ESM.pdf]

Deep learning-based breast cancer diagnosis in breast MRI: Systematic review and meta-analysis

ELECTRONIC SUPPLEMENTARY MATERIAL

SUPPLEMENTAL TEXT AND TABLES

Supplemental Text 1 : Definitions of commonly used terms in this review.

Testing
The evaluation of an algorithm’s performance.

Development
The training, tuning, and validation of an algorithm.

External testing
When an algorithm is tested by an independent third party who has not been involved in the development of the algorithm.

Internal testing
When an algorithm is tested by the company/academic institution that developed it.

External dataset
A dataset that is from a different dataset to the dataset that was used for development (training and validation). This can be either geographically (from a different site or country), temporally (from a different time period), or both geographically and temporally different.

Internal dataset
A dataset that is from the same dataset as the dataset that was used for development (training and validation), which is used for testing.

Convolutional neural networks (CNNs)
A type of deep learning algorithm designed to process structured grid data such as images. CNNs are characterized by their use of convolutional layers that apply a series of filters to input data to extract hierarchical features, making them particularly effective for image recognition tasks.

Hybrid composite models (HCMs)
Advanced AI models that combine different types of neural network architectures, such as Convolutional Neural Networks (CNNs) and Recurrent Neural Networks (RNNs), to leverage the strengths of each in processing both spatial and sequential data. These models are designed to enhance the performance and robustness of AI applications in complex tasks such as medical imaging.

Cross validation or k-fold
A model evaluation method that involves dividing the dataset into k subsets or "folds." The model is trained on k-1 folds and tested on the remaining fold. This process is repeated k times, with each fold being used as the test set once. The average performance across all k trials is used to assess the model's effectiveness, providing a robust estimate of its generalizability.

Segmentation
The process of partitioning an image into multiple segments or regions, often to isolate specific structures or objects of interest. In medical imaging, segmentation is used to delineate anatomical structures or pathological areas, such as tumors, within an image, facilitating detailed analysis and diagnosis.

Supplemental Text 2 : Protocol registration with PROPERO (International Prospective Register of Systematic Review).

PROSPERO (CRD42019156016)

https://www.crd.york.ac.uk/prospero/display_record.php?ID=CRD42024485371

Link to protocol

https://www.crd.york.ac.uk/prospero/export_record_pdf.php

Table S1 : A total of 40 studies have been included. Below are the remaining 19 articles included, in addition to those listed in Table 2.

Authors, Year	Sample Size	Total Data Split (Training, Validation, Testing)			Data Split Ratio (Testing Dataset)	Lesions	Performance Metrics (AUC / Sensitivity / Specificity / Accuracy)	Preprocessing (Category/ Methods)
**Bhowmik et al., 2023**	8274 (patients) 16,535 (images)	training 6564 (patients) 13,081 (images)	validation 855 (patients) 1,687 (images)	testing 855 (patients) 1,687 (images)	8:1:1 (external)	total: 1,382 (cases) benign: 159 (cases) malignant: 59 (cases)	Sensitivity: 1.00 Specificity: 0.12 (0.10-0.13)	UNet for segmentation using MIP (Automatic Segmentation)
**Jing et al., 2023**	488 (patients) 837 (exams) 962 (breasts)	training 257 (breasts)		testing 700 (breasts)	Not stated	total: 265 (lesions) normal: 799 (breasts) benign: 190 (breasts) malignant: 75 (breasts)	Sensitivity: 0.96 (0.87-1.00)	3D UNet for segmentation using T1W prior generating MIP (Automatic Segmentation)
**Antropova et al., 2018**	690 (patients)	training 552 (patients)	validation 138 (patients)		8:1:1 (Not stated)	total: 1,040 (patients) normal: 350 (patients) benign: 212 (patients) malignant: 478 (patients)	AUC: 0.88	ROIs to generate MIP – subtracted (Manual Segmentation)
**Raimundo et al., 2023**	922 (patients) 772,439 (images)	training 866 (patients), 77,963 (images) Slices with lesions: 41,317 (images)		testing 56 (patients) 5,031 (images)	9:1 (train:test) (internal)	Not stated	Accuracy: 0.94	Images resized; XML annotations generated (Cropping and Labeling)
**X. Jing et al., 2022**	122 (patients) 173 (breasts)	Not stated			Not stated (internal)	total: 173 (lesions) benign: 109 (lesions) malignant: 64 (lesions)	AUC: 0.83 Sensitivity: 0.96 (0.95-0.96)	Breast segmentation using 3D U-Net (Automatic Segmentation)
**Xueping Jing et al., 2022**	488 (patients) 837 (exams) 1,674 (breasts)	training 494 (patients) 214 (exams)	validation 165 (patients) 125 (exams)	testing (149 (patients) 178 (exams)	6:2:2 (internal)	Not stated	AUC: 0.81 (0.75-0.88) Sensitivity: 0.98 (0.90-1.00)	Breast segmentation using 3D U-Net (Automatic Segmentation)

**Jiao et al., 2020**	75 (patients)	Not stated			Not stated	Not stated	Sensitivity: 0.87	U-Net++ for breast region segmentation<br><br>(Automatic Segmentation)
**Sun et al., 2023**	1,003 (patients)	training 803 (patients)	validation 100 (patients)	testing 100 (patients)	8:1:1 (internal)	normal: 400 (patients)	AUC: 0.94 (0.92-0.94)<br>Sensitivity: 0.84	Background subtraction; normalization using intensity deviation method<br><br>(Normalization)
**Wu et al., 2022**	130 (patients)	training 78 (patients)	validation 26 (patients)	testing 26 (patients)	Not stated (internal)	total: 130 (lesions)<br>benign: 59 (lesions)<br>malignant: 71 (lesions)	AUC: 0.91 (0.87-0.95)<br>Sensitivity: 0.86 (0.85-0.87)<br>Accuracy: 0.88 (0.86-0.90)	Gaussioan filtering for tumor ROI images<br><br>(Gaussian Filtering)
**Wang et al., 2023**	310 (patients)	training 3,490 (lesions)	validation 430 (lesions)	testing 430 (lesions)	8:1:1 (internal)	total: 4.350 (lesions)<br>benign: 2,124 (lesions)<br>malignant: 2,226 (lesions)	AUC: 0.79<br>Accuracy: 0.98	Cropped images using Photoshop, focusing on breast and axillary tissues<br><br>(Cropping)
**Wang & Wang, 2023**	6,000 (images)	training 4,800 (images)		testing 1,200 (images)	Not stated (internal)	Not stated	Accuracy: 0.98	Converted images into JPEG format<br><br>(Format Conversion)
**Witowski et al., 2022**	13463 (patients)	Not stated			6:1.5:2.5 (external)	Not stated	AUC: 0.97 (0.94-0.99)	Subtraction between post-contrast and pre-contrast images<br><br>(Subtraction)
**Yu et al., 2021**	90 (patients)	training 5,600 (images)	validation 700 (images)	testing 700 (images)	8:1:1 (internal)	total: 7,000 (images)<br>normal: 4,500 (images)	AUC: 0.93<br>Accuracy: 0.95	Normalized images by GCN; random horizontal flips for augmentation, resized to 256×256<br><br>(Normalisation and Augmentation)

**Zhang et al., 2023**	260 (patients)	training 176 (patients)		testing 84 (patients)	Not stated (internal)	total: 176 (patients) benign: 73 (patients) malignant: 103 (patients)	Sensitivity: 0.81 Specificity: 0.81	Segmented left and right breasts; subtraction performed<br><br>(Segmentation and Subtraction)
**Zhou et al., 2020**	Not stated	training 133 (patients)		testing 74 (patients)	Not stated (internal)	benign: 88 (patients) malignant: 139 (patients)	Sensitivity: 0.94 Specificity: 0.81 Accuracy: 0.89	3D tumor segmentation using fuzzy clustering<br><br>(Automatic Segmentation)
**Hu et al., 2020**	616 (patients)	training 394 (patients)	validation 99 (patients)	testing 123 (patients)	Not stated (internal)	total: 927 (lesions) benign: 199 (lesions) malignant: 728 (lesions)	AUC: 0.87 (0.84-0.89)	Fuzzy C-means for lesion segmentation; bicubic interpolation for T2W slices<br><br>(Manual Segmentation)
**Dalmiş et al., 2018**	401 (patients)	training 201 (patients)	validation 40 (patients)	testing 160 (patients)	Not stated (internal)	malignant: 137 (lesions)	Sensitivity: 0.64 (0.60-0.68)	Post-con subtraction volume normalized to pre-con; motion correction using Elastix toolbox<br><br>(Registration)
**Dalmiş et al., 2019**	465 (patients)	Not stated			Not stated (Not stated)	total: 576 (lesions) benign: 208 (lesions) malignant: 368 (lesions)	AUC: 0.85 (0.82-0.89)	3D cubic patches extracted from MRI volumes<br><br>(3D Patch Extraction)
**Ayatollahi et al. 2021**	462 (patients) 489 (exams)	Not stated			Not stated (internal)	total: 572 (lesions) benign: 207 (lesions) malignant: 365 (lesions)	Sensitivity: 0.95 (0.93-0.98)	Normalized patient images to a reference time-point post-contrast and cropped to breast tissue<br><br>(Normalization and Cropping)

Table S2 : QUADAS-2 Tool for the Quality Assessment of Diagnostic Accuracy Studies.

AUTHORS (YEAR)	RISK OF BIAS				APPLICABILITY		
	PATIENT SELECTION	INDEX TEST	REFERENCE STANDARD	FLOW AND TIMING	PATIENT SELECTION	INDEX TEST	REFERENCE STANDARD
Adachi, M., et al. (2020)	Low Risk	Low Risk	Low Risk	Low Risk	Low Risk	Low Risk	Low Risk
Antropova, N., et al. (2018)	Low Risk	Low Risk	Low Risk	Low Risk	Low Risk	Low Risk	Low Risk
Antropova, N., et al. (2019)	High Risk	Low Risk	Low Risk	Low Risk	Low Risk	Low Risk	Low Risk
Bhowmik, A., et al. (2023)	Low Risk	Low Risk	Unclear Risk	Low Risk	Low Risk	Low Risk	Low Risk
Cong, C., et al. (2024)	Low Risk	Low Risk	Unclear Risk	Low Risk	Low Risk	Low Risk	Low Risk
Dalmış, M. U., et al. (2018)	Low Risk	Low Risk	Unclear Risk	Low Risk	Low Risk	Low Risk	Low Risk
Eskreis-Winkler, S., et al. (2021)	Low Risk	Low Risk	Unclear Risk	Low Risk	Low Risk	Low Risk	Unclear Risk
Fan, M., et al. (2023)	Low Risk	Low Risk	Unclear Risk	Low Risk	Low Risk	Low Risk	Low Risk
Hizukuri, A., et al. (2021)	Low Risk	Low Risk	Low Risk	Low Risk	Low Risk	Low Risk	Low Risk
Hu, Q., et al. (2020)	Low Risk	Low Risk	Unclear Risk	Low Risk	Low Risk	Low Risk	Low Risk
Hu, Q., et al. (2021)	Low Risk	Low Risk	Low Risk	Low Risk	Unclear Risk	Low Risk	Unclear Risk
Jiao, H., et al. (2020)	High Risk	Low Risk	Low Risk	Low Risk	Low Risk	Low Risk	Low Risk
Jing, X., et al. (2022)	High Risk	Low Risk	Unclear Risk	Low Risk	Low Risk	Low Risk	Low Risk
Jing, X., et al. (2023)	High Risk	Low Risk	Low Risk	Low Risk	Low Risk	Low Risk	Low Risk
Jing, X., et al. (2022)	Low Risk	Low Risk	Low Risk	Low Risk	Low Risk	Low Risk	Low Risk
Liu, M. Z., et al. (2022)	Low Risk	Low Risk	Low Risk	Low Risk	Low Risk	Low Risk	Low Risk
Parekh, V. S., et al. (2020)	Low Risk	Low Risk	Unclear Risk	Low Risk	Unclear Risk	Low Risk	Low Risk
Raimundo, J. N. C., et al. (2023)	Low Risk	Low Risk	Low Risk	Low Risk	Low Risk	Low Risk	Low Risk
Rasti, R., et al. (2017)	High Risk	Low Risk	Low Risk	Low Risk	Low Risk	Low Risk	Low Risk
Sun, R., et al. (2023)	Low Risk	Low Risk	Low Risk	Low Risk	Low Risk	Low Risk	Low Risk
Tang, W., et al. (2023)	Unclear Risk	Low Risk	Unclear Risk	Low Risk	Unclear Risk	Low Risk	Unclear Risk
Wang, L., et al. (2022)	Low Risk	Low Risk	Low Risk	Low Risk	Low Risk	Low Risk	Low Risk
Wang, L., et al. (2023)	Low Risk	Low Risk	Low Risk	Low Risk	Low Risk	Low Risk	Unclear Risk
Wang, W. and Y. Wang (2023)	High Risk	Low Risk	Low Risk	Low Risk	Low Risk	Low Risk	Low Risk
Witowski, J., et al. (2022)	Low Risk	Low Risk	Low Risk	Low Risk	Low Risk	Low Risk	Low Risk
Wu, Y. A., et al. (2022)	Low Risk	Low Risk	Low Risk	Low Risk	Low Risk	Low Risk	Low Risk
Yin, H., et al. (2021)	Low Risk	Low Risk	Low Risk	Low Risk	Low Risk	Low Risk	Low Risk

Yin, H. L., et al. (2023)	Low Risk	Low Risk	Low Risk	Low Risk	Low Risk	Low Risk	Low Risk
Yu, H., et al. (2021)	Low Risk	Low Risk	Low Risk	Low Risk	Low Risk	Low Risk	Low Risk
Zhang, Y., et al. (2022)	Low Risk	Low Risk	Low Risk	Low Risk	Low Risk	Low Risk	Low Risk
Zhang, Y., et al. (2023)	Low Risk	Low Risk	Low Risk	Low Risk	Low Risk	Low Risk	Low Risk
Zhou, J., et al. (2019)	Low Risk	Low Risk	Unclear Risk	Low Risk	Unclear Risk	Low Risk	Low Risk
Zhou, J., et al. (2020)	Low Risk	Low Risk	Low Risk	Low Risk	Low Risk	Low Risk	Low Risk
Sheng et al. 2021	Low Risk	Low Risk	Low Risk	Low Risk	Low Risk	Low Risk	Low Risk
Dalmiş et al., 2019	High Risk	Low Risk	Low Risk	Low Risk	Low Risk	Low Risk	Low Risk
Gui et al. 2022	Low Risk	Low Risk	Low Risk	Low Risk	Low Risk	Low Risk	Low Risk
Chen et al. 2022	High Risk	Low Risk	Low Risk	Low Risk	Low Risk	Low Risk	Low Risk
Zhu et al. 2022	Low Risk	Low Risk	Low Risk	Low Risk	Low Risk	Low Risk	Low Risk
Ayatollahi et al. 2021	Low Risk	Low Risk	Low Risk	Low Risk	Low Risk	Low Risk	Low Risk
Feng et al. 2020	High Risk	Low Risk	Low Risk	Low Risk	Low Risk	Low Risk	Low Risk

Table S3 : MAIC-10 (Must AI Criteria-10).

AUTHORS (YEAR)	CLINICAL NEED	STUDY DESIGN	SAFETY AND PRIVACY	DATA CURATION	DATA ANNOTATION	DATA PARTITIONING	AI MODEL	ROBUSTNESS	EXPLAINABILITY	TRANSPARENCY	TOTAL SCORE
Adachi, M., et al. (2020)	1	0	1	1	1	1	1	0	1	1	8
Antropova, N., et al. (2018)	1	1	1	1	1	1	1	0	1	0	8
Antropova, N., et al. (2019)	1	1	1	1	1	1	1	0	1	0	8
Bhowmik, A., et al. (2023)	1	1	1	1	1	1	1	1	1	1	10
Cong, C., et al. (2024)	1	1	1	1	1	1	1	1	1	1	10
Dalmış, M. U., et al. (2018)	1	1	0	1	1	1	1	1	1	1	9
Eskreis-Winkler, S., et al. (2021)	1	1	1	1	1	1	1	1	1	1	10
Fan, M., et al. (2023)	1	1	1	1	1	1	1	1	1	1	10
Feng et al. 2020	1	1	0	1	1	1	1	1	1	1	9
Hizukuri, A., et al. (2021)	1	1	0	1	1	1	1	0	1	0	7
Hu, Q., et al. (2020)	1	1	1	1	1	1	1	0	1	0	8
Hu, Q., et al. (2021)	1	1	1	1	1	1	1	0	1	1	9
Jiao, H., et al. (2020)	1	1	1	1	1	1	1	0	1	0	8
Jing, X., et al. (2022)	1	1	1	1	1	1	1	0	1	0	8
Jing, X., et al. (2023)	1	1	1	1	1	1	1	1	1	0	9
Jing, X., et al. (2022)	1	1	1	1	1	1	1	0	1	0	8
Liu, M. Z., et al. (2022)	1	1	1	1	1	1	1	1	1	0	9
Parekh, V. S., et al. (2020)	1	1	1	1	0	1	1	0	1	1	8
Raimundo, J. N. C., et al. (2023)	1	1	1	1	1	1	1	1	1	1	10
Rasti, R., et al. (2017)	1	1	0	1	1	1	1	1	1	0	8
Sun, R., et al. (2023)	1	1	1	1	1	1	1	1	1	1	10
Tang, W., et al. (2023)	1	1	1	1	1	1	1	1	1	1	10
Wang, L., et al. (2022)	1	1	1	1	1	1	1	1	1	1	10
Wang, L., et al. (2023)	1	1	1	1	1	1	1	1	1	1	10
Wang, W. and Y. Wang (2023)	1	1	1	1	1	1	1	1	1	0	9
Witowski, J., et al. (2022)	1	1	1	1	1	1	1	0	1	1	9
Wu, Y. A., et al. (2022)	1	1	0	1	1	1	1	0	1	0	7
Yin, H., et al. (2021)	1	1	1	1	1	1	1	0	1	0	8
Yin, H. L., et al. (2023)	1	1	1	1	1	1	1	1	1	0	9
Yu, H., et al. (2021)	1	1	1	1	1	1	1	1	1	0	9
Zhang, Y., et al. (2022)	1	1	1	1	1	1	1	1	1	0	9
Zhang, Y., et al. (2023)	1	1	1	1	1	1	1	1	1	1	10
Zhou, J., et al. (2019)	1	1	1	1	1	1	1	1	1	0	9
Zhou, J., et al. (2020)	1	1	1	1	1	1	1	1	1	0	9
Sheng et al. 2021	1	1	1	1	1	1	1	0	1	1	9

Dalmiş et al., 2019	1	1	1	1	1	0	1	0	1	1	8
Gui et al. 2022	1	1	1	1	1	1	1	0	1	1	9
Chen et al. 2022	1	1	1	1	1	1	1	0	1	0	8
Zhu et al. 2022	1	1	1	1	0	1	1	0	1	1	8
Ayatollahi et al. 2021	1	1	0	1	1	1	1	0	1	0	7

SUPPLEMENTAL FIGURES

Figure S1 : This forest plot shows the individual and pooled AUC values for the diagnostic accuracy of deep learning algorithms in the detection of breast cancer using MRI in 21 studies. The estimates of the individual studies with the corresponding 95% CIs are represented by the squares and horizontal lines, with the size of the square reflecting the weight of the study in the meta-analysis. The diamond represents the pooled AUC estimate, which indicates high diagnostic efficacy (pooled AUC: 90%; 95% CI: 87–93%). High heterogeneity is observed (I^2 = 86%, $p < 0.01$).

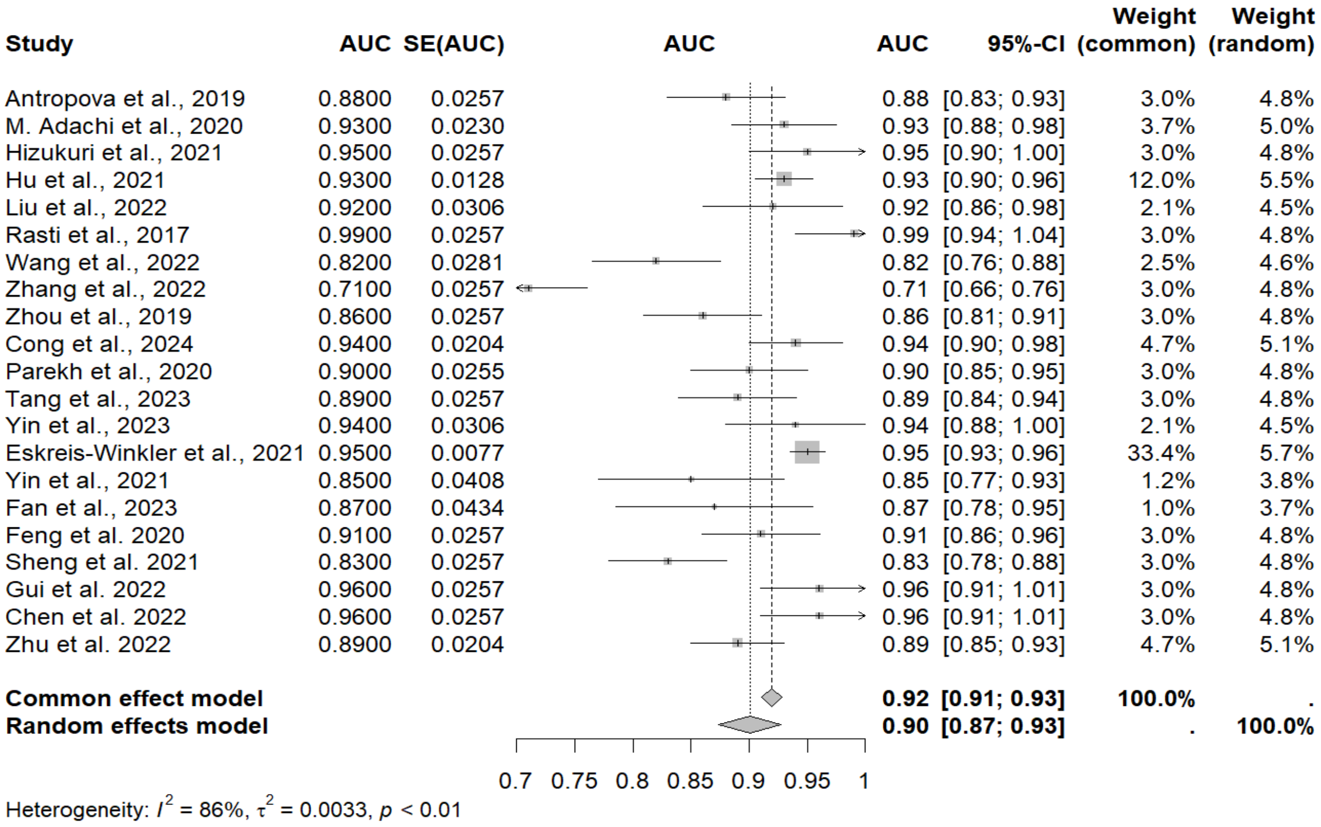

Figure S2 : This figure shows the estimates of the individual studies and the pooled sensitivity for the same group of studies as in Figure 4. The squares represent the estimates of the individual studies, and the size of each square indicates the weight of the study. The horizontal lines show the 95% CIs. The pooled estimate is represented by the diamond at the bottom (pooled sensitivity: 88%; 95% CI: 86–91%), where moderate heterogeneity was observed (I^2 = 55%, $p < 0.01$).

Study	Sensitivity	SE(Sensitivity)	Sensitivity	95%-CI	Weight (common)	Weight (random)
Antropova et al., 2019	0.9000	0.0439	0.90	[0.81; 0.99]	2.9%	4.6%
M. Adachi et al., 2020	0.9300	0.0439	0.93	[0.84; 1.02]	2.9%	4.6%
Hizukuri et al., 2021	0.9300	0.0439	0.93	[0.84; 1.02]	2.9%	4.6%
Hu et al., 2021	0.9400	0.0439	0.94	[0.85; 1.03]	2.9%	4.6%
Liu et al., 2022	0.7400	0.0842	0.74	[0.57; 0.90]	0.8%	2.0%
Rasti et al., 2017	0.9800	0.0439	0.98	[0.89; 1.07]	2.9%	4.6%
Wang et al., 2022	0.8500	0.0459	0.85	[0.76; 0.94]	2.7%	4.4%
Zhang et al., 2022	0.8000	0.0439	0.80	[0.71; 0.89]	2.9%	4.6%
Zhou et al., 2019	0.9100	0.0204	0.91	[0.87; 0.95]	13.5%	7.4%
Cong et al., 2024	0.8900	0.0439	0.89	[0.80; 0.98]	2.9%	4.6%
Parekh et al., 2020	0.8600	0.0439	0.86	[0.77; 0.95]	2.9%	4.6%
Tang et al., 2023	0.8000	0.0439	0.80	[0.71; 0.89]	2.9%	4.6%
Yin et al., 2023	0.9300	0.0439	0.93	[0.84; 1.02]	2.9%	4.6%
Eskreis-Winkler et al., 2021	0.9000	0.0128	0.90	[0.88; 0.92]	34.5%	8.3%
Yin et al., 2021	0.8200	0.0439	0.82	[0.73; 0.91]	2.9%	4.6%
Fan et al., 2023	0.9400	0.0439	0.94	[0.85; 1.03]	2.9%	4.6%
Feng et al. 2020	0.8600	0.0439	0.86	[0.77; 0.95]	2.9%	4.6%
Sheng et al. 2021	0.7900	0.0439	0.79	[0.70; 0.88]	2.9%	4.6%
Gui et al. 2022	0.9500	0.0439	0.95	[0.86; 1.04]	2.9%	4.6%
Chen et al. 2022	0.9600	0.0439	0.96	[0.87; 1.05]	2.9%	4.6%
Zhu et al. 2022	0.7300	0.0561	0.73	[0.62; 0.84]	1.8%	3.6%
**Common effect model**			**0.89**	**[0.88; 0.91]**	**100.0%**	**.**
**Random effects model**			**0.88**	**[0.86; 0.91]**	**.**	**100.0%**

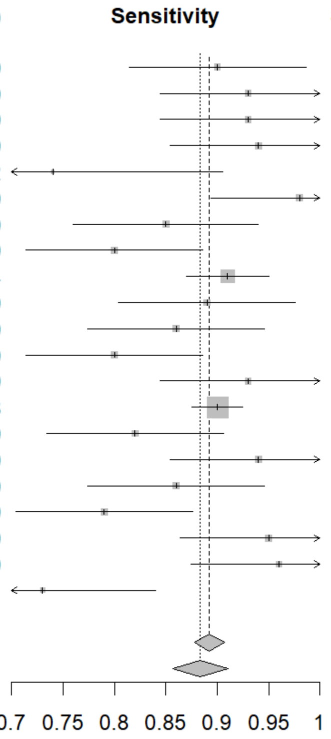

Heterogeneity: I^2 = 55%, τ^2 = 0.0021, p < 0.01

Figure S3 : Here, the forest plot shows the estimates of the individual studies and the pooled specificity for deep learning algorithms in the detection of breast cancer. The study-specific estimates and their 95% CIs are represented by squares and lines respectively, with the size of the squares indicating the weighting of the studies. The diamond symbolises the combined estimate of specificity (pooled specificity: 90%; 95% CI: 87–93%), which shows high heterogeneity between studies ($I^2 = 79\%$, $p < 0.01$).

Study	Specificity	SE(Specificity)	Specificity	95%-CI	Weight (common)	Weight (random)
Antropova et al., 2019	0.8800	0.0316	0.88	[0.82; 0.94]	2.8%	4.8%
M. Adachi et al., 2020	0.9300	0.0316	0.93	[0.87; 0.99]	2.8%	4.8%
Hizukuri et al., 2021	0.9500	0.0316	0.95	[0.89; 1.01]	2.8%	4.8%
Hu et al., 2021	0.9300	0.0316	0.93	[0.87; 0.99]	2.8%	4.8%
Liu et al., 2022	0.9200	0.0281	0.92	[0.86; 0.98]	3.5%	5.0%
Rasti et al., 2017	0.9900	0.0316	0.99	[0.93; 1.05]	2.8%	4.8%
Wang et al., 2022	0.8200	0.0408	0.82	[0.74; 0.90]	1.7%	4.1%
Zhang et al., 2022	0.7100	0.0316	0.71	[0.65; 0.77]	2.8%	4.8%
Zhou et al., 2019	0.8600	0.0459	0.86	[0.77; 0.95]	1.3%	3.7%
Cong et al., 2024	0.9400	0.0316	0.94	[0.88; 1.00]	2.8%	4.8%
Parekh et al., 2020	0.9000	0.0316	0.90	[0.84; 0.96]	2.8%	4.8%
Tang et al., 2023	0.8900	0.0316	0.89	[0.83; 0.95]	2.8%	4.8%
Yin et al., 2023	0.9400	0.0316	0.94	[0.88; 1.00]	2.8%	4.8%
Eskreis-Winkler et al., 2021	0.9500	0.0077	0.95	[0.94; 0.96]	47.2%	6.3%
Yin et al., 2021	0.8500	0.0316	0.85	[0.79; 0.91]	2.8%	4.8%
Fan et al., 2023	0.8700	0.0316	0.87	[0.81; 0.93]	2.8%	4.8%
Feng et al. 2020	0.9100	0.0316	0.91	[0.85; 0.97]	2.8%	4.8%
Sheng et al. 2021	0.8300	0.0316	0.83	[0.77; 0.89]	2.8%	4.8%
Gui et al. 2022	0.9600	0.0316	0.96	[0.90; 1.02]	2.8%	4.8%
Chen et al. 2022	0.9600	0.0316	0.96	[0.90; 1.02]	2.8%	4.8%
Zhu et al. 2022	0.8900	0.0357	0.89	[0.82; 0.96]	2.2%	4.5%
**Common effect model**			**0.92**	**[0.91; 0.93]**	**100.0%**	**.**
**Random effects model**			**0.90**	**[0.87; 0.93]**	**.**	**100.0%**

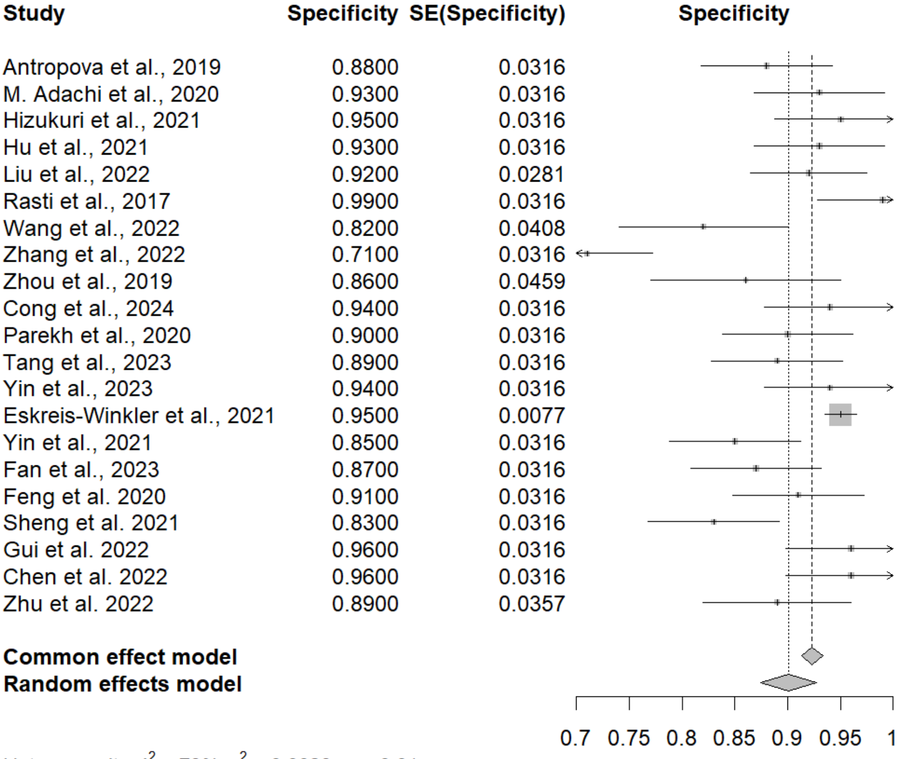

Heterogeneity: $I^2 = 79\%$, $\tau^2 = 0.0029$, $p < 0.01$

Section/topic	#	PRISMA-DTA Checklist Item	Reported on page #
**TITLE / ABSTRACT**			
Title	1	Identify the report as a systematic review (+/- meta-analysis) of diagnostic test accuracy (DTA) studies.	
Abstract	2	Abstract: See PRISMA-DTA for abstracts.	
**INTRODUCTION**			
Rationale	3	Describe the rationale for the review in the context of what is already known.	
Clinical role of index test	D1	State the scientific and clinical background, including the intended use and clinical role of the index test, and if applicable, the rationale for minimally acceptable test accuracy (or minimum difference in accuracy for comparative design).	
Objectives	4	Provide an explicit statement of question(s) being addressed in terms of participants, index test(s), and target condition(s).	
**METHODS**			
Protocol and registration	5	Indicate if a review protocol exists, if and where it can be accessed (e.g., Web address), and, if available, provide registration information including registration number.	
Eligibility criteria	6	Specify study characteristics (participants, setting, index test(s), reference standard(s), target condition(s), and study design) and report characteristics (e.g., years considered, language, publication status) used as criteria for eligibility, giving rationale.	
Information sources	7	Describe all information sources (e.g., databases with dates of coverage, contact with study authors to identify additional studies) in the search and date last searched.	

Search	8	Present full search strategies for all electronic databases and other sources searched, including any limits used, such that they could be repeated.	
Study selection	9	State the process for selecting studies (i.e., screening, eligibility, included in systematic review, and, if applicable, included in the meta-analysis).	
Data collection process	10	Describe method of data extraction from reports (e.g., piloted forms, independently, in duplicate) and any processes for obtaining and confirming data from investigators.	
Definitions for data extraction	11	Provide definitions used in data extraction and classifications of target condition(s), index test(s), reference standard(s) and other characteristics (e.g. study design, clinical setting).	
Risk of bias and applicability	12	Describe methods used for assessing risk of bias in individual studies and concerns regarding the applicability to the review question.	
Diagnostic accuracy measures	13	State the principal diagnostic accuracy measure(s) reported (e.g. sensitivity, specificity) and state the unit of assessment (e.g. per-patient, per-lesion).	
Synthesis of results	14	Describe methods of handling data, combining results of studies and describing variability between studies. This could include, but is not limited to: a) handling of multiple definitions of target condition. b) handling of multiple thresholds of test positivity, c) handling multiple index test readers, d) handling of indeterminate test results, e) grouping and comparing tests, f) handling of different reference standards	

**Section/topic**	**#**	**PRISMA-DTA Checklist Item**	**Reported on page #**
Meta-analysis	D2	Report the statistical methods used for meta-analyses, if performed.	

Additional analyses	16	Describe methods of additional analyses (e.g., sensitivity or subgroup analyses, meta-regression), if done, indicating which were pre-specified.	
**RESULTS**			
Study selection	17	Provide numbers of studies screened, assessed for eligibility, included in the review (and included in meta-analysis, if applicable) with reasons for exclusions at each stage, ideally with a flow diagram.	
Study characteristics	18	For each included study provide citations and present key characteristics including: a) participant characteristics (presentation, prior testing), b) clinical setting, c) study design, d) target condition definition, e) index test, f) reference standard, g) sample size, h) funding sources	
Risk of bias and applicability	19	Present evaluation of risk of bias and concerns regarding applicability for each study.	
Results of individual studies	20	For each analysis in each study (e.g. unique combination of index test, reference standard, and positivity threshold) report 2x2 data (TP, FP, FN, TN) with estimates of diagnostic accuracy and confidence intervals, ideally with a forest or receiver operator characteristic (ROC) plot.	
Synthesis of results	21	Describe test accuracy, including variability; if meta-analysis was done, include results and confidence intervals.	
Additional analysis	23	Give results of additional analyses, if done (e.g., sensitivity or subgroup analyses, meta-regression; analysis of index test: failure rates, proportion of inconclusive results, adverse events).	
**DISCUSSION**			
Summary of evidence	24	Summarize the main findings including the strength of evidence.	
Limitations	25	Discuss limitations from included studies (e.g. risk of bias and concerns regarding applicability) and from the review process (e.g. incomplete retrieval of identified research).	

Conclusions	26	Provide a general interpretation of the results in the context of other evidence. Discuss implications for future research and clinical practice (e.g. the intended use and clinical role of the index test).	
**FUNDING**			
Funding	27	For the systematic review, describe the sources of funding and other support and the role of the funders.	

Adapted From: McInnes MDF, Moher D, Thombs BD, McGrath TA, Bossuyt PM, The PRISMA-DTA Group (2018). Preferred Reporting Items for a Systematic Review and Meta-analysis of Diagnostic Test Accuracy Studies: The PRISMA-DTA Statement. JAMA. 2018 Jan 23;319(4):388-396. doi: 10.1001/jama.2017.19163.

For more information, visit: **www.prisma-statement.org**.

Page 2 of 2
